# Electroacupuncture Suppresses Discrete Cue-Evoked Heroin-Seeking and Fos Protein Expression in the Nucleus Accumbens Core in Rats

**DOI:** 10.1155/2012/286404

**Published:** 2012-02-14

**Authors:** Sheng Liu, Fenglei Zhu, Miaojun Lai, Limin Sun, Yijun Liu, Wenhua Zhou

**Affiliations:** ^1^Ningbo Addiction Research and Treatment Center, School of Medicine, Ningbo University, Ningbo 315010, China; ^2^Laboratory of Acupuncture and Moxibustion, Shanghai University of Traditional Chinese Medicine, Shanghai 200032, China; ^3^Laboratory of Neuroscience, 81 Brain Hospital, Beijing 100700, China; ^4^Laboratory of Behavioral Neuroscience, Ningbo Addiction Research and Treatment Center, School of Medicine, Ningbo University, Ningbo 315010, China

## Abstract

Relapse to drug seeking was studied using a rodent model of reinstatement induced by exposure to drug-related cues. Here, we used intravenous drug self-administration procedures in rats to further investigate the beneficial effects of electroacupuncture (EA) on heroin-seeking behavior in a reinstatement model of relapse. We trained Sprague-Dawley rats to nose-poke for i.v. heroin either daily for 4 h or 25 infusions for 14 consecutive days. Then the rats were abstinent from heroin for two weeks. 2 Hz EA stimulation was conducted once daily for 14 days during heroin abstinence. We tested these animals for contextual and discrete cue-induced reinstatement of active responses. We also applied immunohistochemistry to detect Fos-positive nuclei in the nucleus accumbens (NACc) core and shell after reinstatement test. We found that active responses elicited by both contextual cues and discrete cues were high in the rats trained with heroin than in saline controls. EA treatment significantly reduced active responses elicited by discrete cues. EA stimulation attenuated Fos expression in the core but not the shell of the NACc. Altogether, these results highlight the therapeutic benefit of EA in preventing relapse to drug addiction.

## 1. Introduction

Drug addiction is characterized by relapse to drug-seeking behavior during periods of abstinence [1]. Acupuncture and electroacupuncture (EA) have been applied with great success to attenuate various conditions related to drug addiction [2, 3]. In animal model, EA significantly attenuates morphine-induced conditioned place preference (CPP) and behavioral sensitization [4–6]. Recently, Yang et al. [7] and Yoon et al. [8] reported that acupuncture can suppress morphine and ethanol self-administration. Similarly, using the self-administration model of reinstatement, we found that EA attenuates the reinstatement of heroin-seeking behaviors induced by heroin priming [9]. These findings provided new evidence that EA or acupuncture might have therapeutic effect on drug-seeking behaviors.

 One factor that contributes to drug-seeking behavior is the presence of cues and contexts that it previously has associated with past drug use. The motivational effect of cue presentation is illustrated most dramatically by the reports of drug craving and relapse to drug-seeking behavior by addicts while in the presence of drug-associated cues and contexts [10–13]. In laboratory animals, discrete conditioned stimuli (cues) (e.g., tone, light, and sound of infusion pump) or contextual conditioned stimuli (cues) (e.g., operant chamber fan and time of day) reinstate drug seeking after extinction of the drug-taking behavior in the absence of these cues [14, 15]. The experiments reported here designed to further examine the effects of EA on discrete or contextual cue-induced reinstatement of heroin-seeking behavior after heroin self-administration.

The neural circuitry of cue-elicited drug seeking involves the nucleus accumbens (NACc) [16, 17]. Exposure to drug-paired cue causes an elevation in NACc neuron firing rates [17] and extracellular dopamine (DA) levels [16], as well as increased levels of c-Fos, a marker for neuronal activation [18]. Interestingly, Yoon et al. [8] reported that acupuncture stimulation at bilateral Shenmen (HT7) attenuated c-fos expression in the NACc utilizing the immunocytochemical detection of Fos protein in nicotine-sensitized rats. Furthermore, acupuncture significantly decreased both dopamine release in the NACc and behavioral hyperactivity induced by a systemic morphine challenge [6]. These findings suggest that acupuncture produces a therapeutic effect on opioid or nicotine addiction, possibly by modulating postsynaptic neuronal activity in the NACc (for review, see [19]). Given that there are anatomical differences between NACc core and shell in both neuronal morphology and connectivity, and these subregions play different roles in drug seeking [20–22], we also applied Fos immunomapping to investigate different functional activation of the NACc core and shell after cue-induced reinstatement of heroin-seeking and EA stimulation.

## 2. Methods

### 2.1. Subjects

Male Sprague-Dawley rats (250–300 g) from the Zhejiang Center of Experimental Animals were used. They were randomly assigned and housed collectively (four per cage) under controlled environmental conditions (22°C, 12-h light/dark cycle) with free access to food and water. All animal treatments were performed in strict accordance with the National Institutes of Health Guide for the Care and Use of Laboratory Animals. All experiments were conducted during the light cycle.

### 2.2. Surgical Procedure

The animals were implanted with chronically indwelling intravenous catheters under sodium pentobarbital (50 mg/kg, ip) anesthesia. A silicon catheter (3.5 cm length, 0.5 mm inner diameter, 0.94 mm outer diameter) was inserted into the right external jugular vein and secured with thread so that the tip reached the right atrium. The other end of the catheter (10 cm length, PE20) exited from an incision on the back of the body. The catheters were flushed daily with 0.2 mL saline containing sterile benzylpenicillin sodium (60,000 units) and heparin (5 units), to prevent bacterial infection and maintain catheter patency and capped daily. All the animals were allowed to recover for at least 7 days. The rats were housed individually in stainless-steel mesh home cages (size 25 × 30 × 30 cm) after surgical procedure.

### 2.3. Self-Administration Apparatus

Training and testing were conducted in stainless-steel operant chambers (size 30 × 30 × 30 cm) placed in a sound-attenuated, temperature-controlled room; the light of the room was turned off during training. The apparatus consisted of 24 chambers equipped with two nose-pokes (ENV-114 M, Med Associates, Lafayette, IN) in the back wall. There were three LED lights (green, red, and yellow) inside each nose-poke hole. A cue-light (28 V, 0.1 mA, ENV-215 M, Med Associates) was situated on the wall above the nose-pokes. Drug solution was delivered through Tygon tubing, protected by a leash assembly (PHM-120, Med Associates), and suspended through the ceiling of the chamber from a plastic fluid swivel (PHM-115, Med Associates). The leash assembly was modified to fit a custom-made fluid connector fixed with an animal jacket. The Tygon tubing was attached to a syringe pump (PHM-100, Med Associates) that delivered fluid at a speed of 1.08 mL/min using a 10-mL syringe. The experimental events were controlled by an IBM-compatible PC using a MED Associates interface, running self-programmed software (OBSM v4.0, operant behavioral schedule manager) written in Borland Delphi 6.0. Heroin was obtained from the Institute of Forensic Science, Ministry of Public Security of the People's Republic of China and dissolved in physiological saline.

### 2.4. Self-Administration Procedure and Conditioning Protocols

All the rats underwent an identical sequence of behavioral training. Each rat was trained with one daily 4 h session for 14 consecutive days with either saline or heroin self-administration. The animals were transferred into the operant chambers before each training session and were put back in their individual home cages after the session where food was available. Water was always available both in the test cages and home cages. The rats were not placed in food restriction schedule. Enough food was provided to maintain natural weight gain.

The reinforcement schedule was a modified progressive ratio schedule that involved incrementing response requirements in a relatively gradual manner. The response requirement increased in a linear pattern as calculated according to the following equation: response requirement = truncate (0.2*(step-1) +1), where the results were truncated to integer value. The step number is the number of ratios completed. So in each daily session, the response requirements were 1 for the first five heroin infusions, 2 for the second five infusions, 3 for the third five infusions, 4 for the fourth five infusions, and 5 for the last five infusions. Based on our preliminary experiment, this schedule supported reliable heroin self-administration across a range of heroin doses, and the response training was as easy as a FR1 schedule but maintained a relatively high rate of responding, so the schedule could be kept constant throughout the sessions.

Each trial began with illumination of a green light inside the active nose-poke hole. Responding in the active hole resulted in an infusion of heroin (0.05 mg/kg) delivered by an infusion pump (PHM-100, Med Associates, Lafayette, IN). The green nose-poke light was turned off during heroin infusions. A 30 s intertrial interval (time out) followed and then another trial began. Responding in the inactive hole had no consequences. The response requirements started with one and increased one after each five heroin infusion. Each earned heroin infusion was also paired with a 5 s cue-light (situated on the wall above the nose-pokes) that served as the discrete cue stimulus. The session ended after 25 infusions were earned or 4 hours had passed, whichever came first.

### 2.5. Abstinence and Reinstatement

After 14 days of self-administration training, the rats were made to abstain from heroin for another 14 days during which they were confined to their individual home cages. The choice of abstinence duration was based on our previous work [23]. Using the same experimental procedure, we found that there were no significant differences of discrete CS-induced heroin seeking among the rats after 1, 2, and 4 weeks abstinence from self-administration.

The reinstatement testing lasted for 2 hours, and each animal was tested only once. During testing, the animals still wore their jackets, but the leash assemblies were not connected. The testing consisted of two consecutive 1-h phases. During the first phase, the rats were allowed to respond to the nose-pokes with all the conditioned stimulus lights kept off. The responses were recorded. This phase was used to measure contextual cue- (chamber environment) induced heroin seeking and was generally regarded as an extinction phase.

Immediately after the first phase, the second phase began. This was signaled by one 5 s presentation of the discrete cue stimulus (the nose-poke light, the cue light, and the pump noise), which was previously paired with each heroin infusion. During this phase, the green light inside the active nose-poke hole was turned on, and each active response resulted in another 5 s presentation of the discrete cue stimulus and the turning off of the green nose-poke light. After turning off the discrete cue stimulus, another trial began. This phase was used to measure discrete cue-induced heroin seeking.

### 2.6. EA Stimulation

Rats were kept in special holders with their hind legs and tails exposed. Two stainless steel needles of 0.3 mm diameter were inserted into each hind leg in the acupoints ST36 (5 mm lateral to the anterior tubercle of the tibia) and SP6 (3 mm proximal to the superior border of the medial malleolus, at the posterior border of the tibia). Constant current squarewave electric stimulation produced by an electroacupuncture apparatus (Model G-6805-2, Shanghai Medical Electronic Apparatus, China) was administered via the two needles. The frequency of stimulation used was 2 Hz. The intensity of the stimulation was increased stepwise from 0.5 to 1.0 mA, with each step lasting for 15 min.

### 2.7. Fos Immunohistochemistry

Four rats from each group were randomly selected for c-Fos immunohistochemistry. Immediately after behavioral testing, rats were deeply anesthetized with sodium pentobarbital (60 mg/kg, ip) and killed by transcardial perfusion of 200 mL ice saline followed by 200 mL 4% paraformaldehyde in 0.1 mol/L phosphate buffer (PB). Brains were dissected and postfixed in the same fixative and then stored in 30% sucrose at 4°C for 3–5 days. Coronal sections (30 *μ*m in thickness; 1.6 mm from bregma according to the atlas of Paxinos and Watson [24]) were cut on the cryostat at −25°C. Sections were rinsed in 0.01 M phosphate-buffered saline (PBS) and incubated in PBS containing 5% normal goat serum and 0.3% Triton X-100 for 30 min and then in Fos antibody (rabbit polyclonal antibody. Santa Cruz, USA) diluted at 1 : 200 in PBS at 4°C for 48 h. After rinsing three times with PBS, sections were incubated in the biotinylated goat anti-rabbit secondary antibody (Sigma, USA, diluted 1 : 200 with PBS) for 2 h and washed again. Then all corresponding sections were placed in the avidin-biotin-peroxidase complex solution for 60 min. Finally, DAB was used for visualization of Fos immunoreactivity. The reaction was stopped by several PBS washes. Sections were then mounted on gelatin-coated slides, air-dried, dehydrated through graded alcohols, cleared in xylene, and coverslipped with Eukitt. 

### 2.8. Quantification of Fos-Positive Nuclei in the NACc

The NACc is an integral part of the basal ganglia located within the ventral striatum. It is composed of two regions: core and shell (see Figure 4(f)). Sections were scanned using an Olympus BX51 microscope. Image analysis was carried out with the aid of an image analysis system (40x magnification). Three consecutive sections were taken from each animal, and the Fos-positive nuclei were counted bilaterally, based on a randomization procedure. A computer-generated rectangle (250 × 600 um) was placed in a fixed area of the NACc core and shell of each section, and the analysis software counted stained nuclei within the area.

### 2.9. Experimental Protocols

Forty rats were trained with heroin self-administration. Training sessions were conducted daily for 14 consecutive days. Then the rats were abstinent from heroin for two weeks, during which they lived in their individual home cages. The heroin-trained rats were divided randomly into four groups: the contextual-cue-induced reinstatement (No EA CONT, *n* = 10), contextual/discrete-cue-induced reinstatement (No EA CONT/DIS, *n* = 10), EA CONT/DIS (*n* = 10), and restraint CONT/DIS (kept in the special holders, *n* = 10). The same experimental procedures were used for the control (No EA SAL, *n* = 6) rats except the heroin was substituted with the same volume of saline. EA (or restraint) treatment was given once daily for 14 days during heroin abstinence. No EA CONT group was allowed to nose-poke for 2 h with all the light signals off. It was used to measure contextual cue- (chamber environment) induced heroin seeking. The No EA CONT/DIS, EA CONT/DIS, restraint CONT/DIS, and No EA SAL rats were allowed to nose-poke for two consecutive 1 h testing phases. All the light signals were turned off in the first phase, and discrete cue stimuli were presented in the second phase.

### 2.10. Statistical Analysis

Experimental data were expressed as mean ± SEM. The differences in total active responses and heroin infusions during heroin self-administration were analyzed using two-way analysis of variance (ANOVA) with session as a repeated within-subject factor and group as a between-subject factor. Cue-induced active responding during reinstatement testing was also analyzed using two-factor repeated ANOVA with time block (15 min) as a within subject factor and group as a between-subject factor. Significant effects were followed by post hoc Tukey tests. Fos protein expression was analyzed using one-way ANOVA. When significance was found using ANOVA procedures, post hoc analyses were conducted using Fisher LSD test. *P* < 0.05 was considered statistically significant.

## 3. Results

### 3.1. Heroin Self-Administration

**Figure 1:**
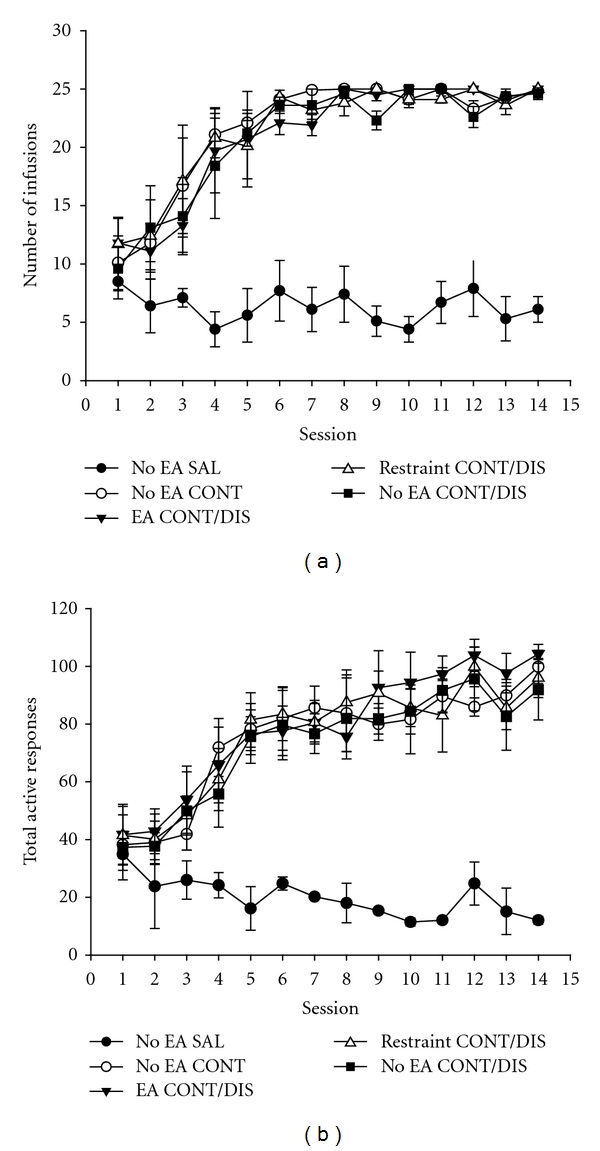
Acquisition of intravenous heroin self-administration. Data were expressed with mean ± S.E.M. of total infusions (a) and total active nose-poke responses (b) during each daily session.

As shown in Figure 1, all heroin-trained rats demonstrated reliable heroin self-administration, as indicated by the increase in infusions and active responses and reached stable levels of heroin infusions and active responses within 14 days of heroin self-administration training without there being any differences between groups in total active responses and infusions per session. This was reflected by the nonsignificant main effects of heroin groups (*F*(3, 36) = 1.29, *F*(3, 36) = 0.83, resp., for total responses and infusions per session, NS), interactions between heroin groups and training sessions (*F*(39, 468) = 1.46, *F*(39, 468) = 0.65, resp., for total responses and infusions per session, NS), and the significant main effect of training sessions (*F*(13, 468) = 53.92, *F*(13, 468) = 42.74, resp., for total responses and infusions per session; *P* < 0.001) (Figures 1(a) and 1(b)). Tukey post hoc test comparisons revealed that rats quickly learned to self-administer heroin such that a significantly higher number of the active responses and infusions were observed at the fourth training session (*P* < 0.001) with stable heroin self-administered behavior acquired after the sixth session. The rats that were trained with saline could not establish stable self-administration; just a few infusions were made within each session (Figure 1). Only minimal responding was observed at the inactive nose-poke for all the training groups and during all the training sessions (data not shown). 

### 3.2. Effect of EA on Cue-Elicited Drug Seeking Behavior

**Figure 2:**
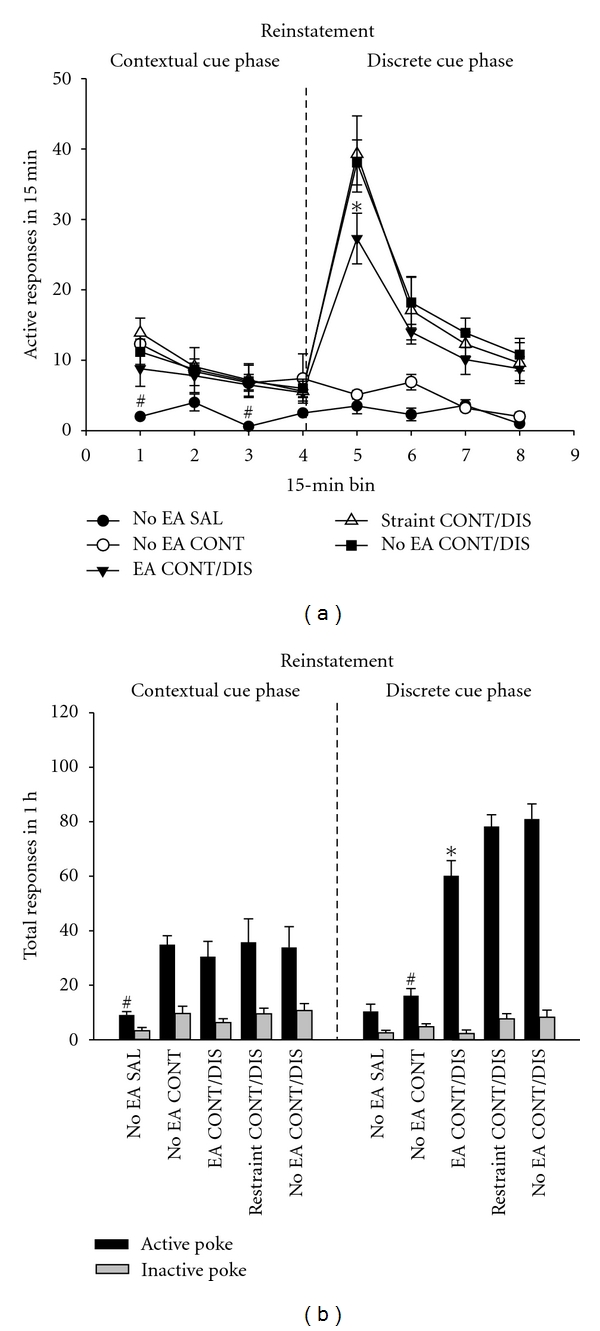
Effect of EA treatment on cue-induced heroin-seeking after two weeks of abstinence from heroin self-administration. Data were expressed with mean ± S.E.M. Number of active response rates in 15 min blocks (a) or total responses (b) in contextual cue phase and discrete cue phase. *P < 0.05 indicates a difference from No EA CONT/DIS group and Restraint CONT/DIS group. ^#^ *P* < 0.01 indicates a difference from No EA CONT/DIS, restraint CONT/DIS, No EA CONT, and EA CONT/DIS group.

After 14 days of abstinence from heroin self administration, the rats were returned to the operant chambers for testing cue-evoked heroin-seeking behavior. Two-factor ANOVA revealed significant main effects of block (*F*(3, 123) = 14.31, *F*(3, 123) = 43.20, resp., for contextual and discrete cues, *P* < 0.001), group (*F*(4, 41) = 12.34, *F*(4, 41) = 35.13, resp., for contextual and discrete cues, *P* < 0.001), and also a significant interaction between block and group (*F*(12, 123) = 6.10, *F*(12, 123) = 11.90, resp., for contextual and discrete cues, *P* < 0.01). As shown in Figure 2(a), heroin cue-induced reinstatement of active responding occurred mainly in the first 15 min blocks and decreased across blocks during both contextual and discrete cue-induced reinstatement testing phases. In contextual cue phase, Tukey post hoc test comparisons revealed active responses were higher in No EA CONT/DIS, EA CONT/DIS, restraint CONT/DIS, and No EA CONT group than in No EA SAL group in the first and the third block (all *P* < 0.01). There were no significant differences among No EA CONT/DIS, EA CONT/DIS, restraint CONT/DIS, and No EA CONT group, although there was a trend for EA to decrease active responses (*P* > 0.05). In discrete cue phase, EA produced significant reductions in active responses in the first 15 min block (*P* < 0.05), as compared with No EA CONT/DIS and Restraint CONT/DIS group.

When the total amount of active responses were analyzed, one-way ANOVA revealed significant effects of group for both contextual cues (*F*(4, 41) = 11.76, *P* < 0.01) and discrete cues (*F*(4, 41) = 26.37, *P* < 0.001) testing phases (Figure 2(b)). In contextual cue phase, total active responses were significantly higher in No EA CONT/DIS, EA CONT/DIS, restraint CONT/DIS, and No EA CONT group than in No EA SAL group (all *P* < 0.01). In discrete cue phase, the total amount of active responses was higher in No EA CONT/DIS and Restraint CONT/DIS group than in EA CONT/DIS and No EA SAL group (all *P* < 0.05). Active responses were also higher in EA CONT/DIS group than in No EA CONT group (*P* < 0.05). There were no significant differences between No EA CONT/DIS and restraint CONT/DIS group (*P* > 0.05). Responding at the inactive nose-poke was minimal during both testing phases (Figure 2(b)).

### 3.3. Effect of EA on Fos Protein Expression in the NACc

**Figure 3:**
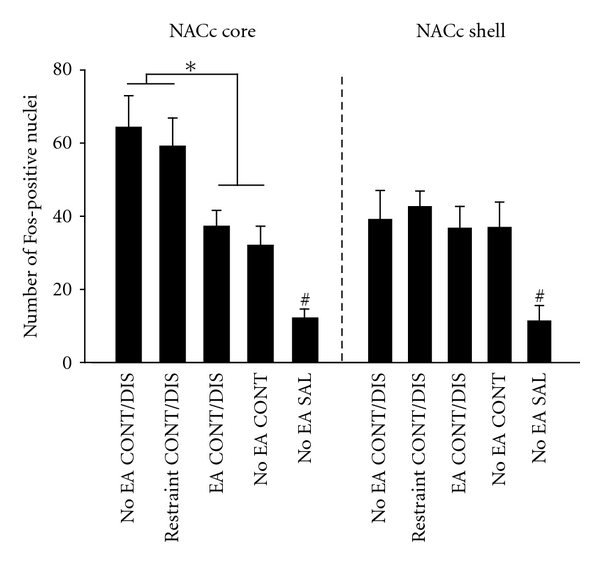
Quantitative analysis of Fos-positive nuclei in the NACc core and shell. Data are expressed as mean ± SEM. *P < 0.05; ^#^ *P* < 0.01 versus No EA CONT/DIS, Restraint CONT/DIS, No EA CONT and EA CONT/DIS group.

**Figure 4:**
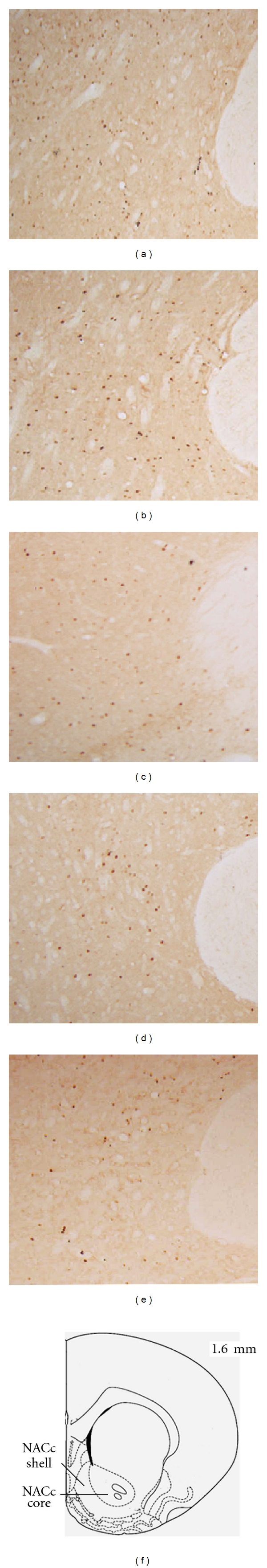
Representative coronal sections showing Fos immunoreactivity in the NACc core. No EA CONT/DIS group (a), restraint CONT/DIS group (b), EA CONT/DIS group (c), No EA CONT group (d), and No EA SAL group (e). Schematic coronal section analyzed. (f) Number at the top right of Figure 4(f) represents the distance (in millimeters) from bregma. The depicted coronal section was adapted from the atlas by Paxinos and Watson [24]. Scale bar, 200 *μ*m.

In the NACc core, One-way ANOVA revealed significant effects of group for Fos protein expression (*F*(4, 55) = 78.21, *P* < 0.001). As illustrated in Figures 3 and 4, only a few Fos positive neurons were detected in No EA SAL group (Figure 4(e)). Compared with No EA CONT group, No EA CONT/DIS and restraint CONT/DIS group exhibited an increase in Fos-positive nuclei (Fisher LSD test; *P* < 0.01). Of note, EA stimulation attenuated Fos expression relative to No EA CONT/DIS and restraint CONT/DIS group (Fisher LSD test; *P* < 0.05). No differences in Fos expression were noted between the No EA CONT/DIS group and Restraint CONT/DIS group (*P* > 0.05).

In the NACc shell, enhanced Fos protein expression was observed in No EA CONT/DIS, EA CONT/DIS, restraint CONT/DIS, and No EA CONT group relative to No EA SAL group. There were no differences in Fos expression among No EA CONT/DIS, EA CONT/DIS, restraint CONT/DIS, and No EA CONT rats, as determined by Fisher LSD post hoc test (*P* > 0.05).

## 4. Discussion

Exposure to environmental stimuli previously associated with drug intake can provoke drug relapse in humans. Environmental cues repeatedly associated with the subjective effects of heroin can elicit drug craving and, possibly, automatic behavioral responses that may lead to relapse in recovering heroin addicts [25–28]. In laboratory animals, discrete-conditioned stimuli that are explicitly paired with opiate injections and contextual-conditioned stimuli that are associated with a distinct opiate environment can reinstate opiate seeking after extinction of the drug-taking behavior in the absence of these cues [27]. Consistent with the mounting evidence suggesting the role of discrete and contextual cue in precipitating relapse [26, 29], our results further confirmed that self-administration environment (contextual cue) and discrete cue stimuli previously associated with heroin injections could elicit robust heroin-seeking behavior after 2-week heroin withdrawal.

In the present study, we found that 2 Hz EA attenuated discrete but not contextual cue-induced reinstatement of heroin seeking after heroin abstinence. These results demonstrate dissociable roles of EA in discrete versus contextual cue-induced reinstatement of heroin seeking. Discrete cues are different from contextual cues. In discrete cue appetitive Pavlovian conditioning, discrete cues with a defined onset and offset that typically activate one sensory modality are provided, accompanied by heroin delivery [30]. Discrete cue functions as a conditioned reinforcer during testing. Associative learning and memory processes may be involved in acquisition and maintenance of associations between drug and drug-paired discrete cues [18, 31]. In contrast, contextual cue is delivered as the animal explores the environment; thus, the temporal relationship between contextual stimuli and reinforcement is not an essential component of the learned associations [32, 33]. Some studies suggest that contextual cue induces heroin seeking by acquiring motivational properties via its direct association with heroin reward during training, independent of its learned associations with the discrete conditional stimuli [34, 35]. This motivational account of contextual cue-induced reinstatement of heroin seeking is supported by the finding that this effect involves the VTA, a brain area involved in the incentive motivational effects of drug and nondrug reinforcers [31]. Based on the above discussion, our current findings seem to suggest that the effects of EA on heroin seeking may be mediated partially by regulating associative learning and memory between drug and drug-paired cues but not motivational properties of drug seeking. Of course, further studies must be performed to clarify this issue.

The NACc core and shell subregions are differentially involved in the reinstatement of cocaine seeking, depending on the type of trigger that elicits this behavior. Our present results that discrete cue evoked an increase in Fos-positive nuclei in the NACc core are consistent with data from several studies using cocaine-trained rats [36]. Fuchs et al. [22] found that reversible inactivation (muscimol plus baclofen) of the NACc core attenuated cocaine seeking in cue-induced reinstatement, and NACc shell inactivation failed to alter cocaine seeking. Similarly, inactivation of the NACc core with muscimol-plus baclofen injection blocked cue-induced reinstatement of cocaine seeking, but inactivation of the NACc shell increaseds cue-induced reinstatement of the extinguished response that had previously delivered cocaine [37]. Moreover, inhibition of NACc core but not shell p70s6k and rps6 phosphorylation decreased discrete cue-induced reinstatement of cocaine seeking [38]. Di Ciano and Everitt reported that permanent lesions or antagonism of AMPA receptors in the core but not the shell decrease discrete cue-induced cocaine seeking, as assessed in a second-order reinforcement schedule [39]. Together, our results and those reviewed above suggest that activation of NACc core neurons mediates discrete cue-induced drug seeking. Our results that EA stimulation suppressed elevated Fos expression in the core but not the shell suggested that the effects of EA on discrete cue-evoked heroin seeking were mediated by suppressing neuronal hyperexcitability in the NACc core. Of note, we also found that contextual cue evoked an increase in Fos-positive nuclei in the NACc shell. It seems to indicate that activation of NACc shell neurons mediates context-induced drug seeking. Such a notion is consistent with the finding that injections of the dopamine D1 receptor antagonist SCH 23390 into the lateral or medial shell, but not core, decreased context-induced reinstatement of heroin seeking [40]. Another study showed that medial shell injections of the metabotropic glutamate 2/3 receptor agonist decreased context-induced reinstatement of heroin seeking [41]. Harris and Aston-Jones [42] reported that Fos induction after exposure to morphine-paired contexts is more pronounced in the shell than in the core. However, another study showed that GABA agonist-induced neural inhibition within the NACc core or shell disrupted context induced reinstatement of cocaine seeking [43]. Moreover, exposure to discriminative cues that predicted cocaine availability increased neuronal activity in the shell but not core [44]. Altogether, these results demonstrate that the NACc is a functionally heterogeneous structure with respect to its involvement in discrete cue- and context-induced cocaine seeking. In present study, we did not observe that EA stimulation reduced elevated Fos expression in the NACc shell. However, Kim et al. [6] demonstrated that acupuncture could decrease the DA release in NACc shell and core. Other studies indicated that the changes of endogenous opioids produced by EA were only observed in NACc shell [45, 46]. The discrepancy between these studies and the present one might be explained by differences in condition of animals and experimental protocols. Numerous examples reveal that the regulatory action of acupuncture is bidirectional. Its therapeutic actions are achieved by normalizing metabolism or pathogenic changes toward homeostasis. For example, differences in intensity and duration of acupuncture stimulation can lead to variation in the induced effects through different nervous pathways. Weak stimulation affects A-beta nerve fibers, whereas strong stimulation affects C fibers [47]. The specific direction of the acupuncture effect may depend on an appropriate selection of certain acupuncture points and variation in technique. For example, contrary to expectations and the results of prior research, Facchinetti et al. [48] demonstrated reduced beta endorphin levels with acupuncture treatment of primary headaches.

Despite knowledge of neuronal activation in the NACc core underlying EA's effectiveness in discrete cue-elicited drug seeking, little is known about neurobiological mechanisms by which EA stimulation exerts a positive influence on drug-seeking behavior. It is possible that acupuncture reduces discrete cue-induced drug-seeking behavior by modulating dopamine release or activation of postsynaptic dopamine receptors in the NACc core. Kim et al. [6] evaluated the effect of acupuncture on repeated morphine-induced changes in extracellular dopamine levels using in vivo microdialysis and repeated morphine-induced behavioral changes. They found that acupuncture significantly decreased both dopamine release in the NACc and behavioral hyperactivity induced by a systemic morphine challenge. These results suggest that the therapeutic effect of acupuncture on morphine addiction occurs through inhibition of drug-induced elevation in dopamine levels in the NACc. On the other hand, 2 Hz EA stimulation was used in the present study. It has been demonstrated that the 2 Hz EA could stimulate the release of endogenous opioid peptide enkephalins and endomorphin in CNS, which interact with mu and delta opioid receptors [46, 49]. The mu and delta opioid receptors are involved in the inhibitory effects of 2 Hz EA on morphine-induced CPP [5]. Moreover, recent studies have shown that EA at 2 Hz increased preproenkephalin mRNA levels in the NACc of morphine CPP rats when the morphine-induced CPP was attenuated by 2 Hz EA [45]. Hence, it is likely that the endogenous opioids and their interaction with mu and delta receptors in the NACc might be involved in the therapeutic benefit of 2 Hz EA in discrete cue-induced drug seeking-behavior.

With regard to Experimental protocols in the present study, there are some variable factors that need to be taken into account. Firstly, it is still controversial as to how to set suitable control group for acupuncture or EA. In order to exclude interference from restraint stress, we run restraint group as a control. However, the perfect control, of course, is one in which needles are inserted into acupuncture points but without electric stimulation. Secondly, in our experimental protocol, the animals tested contextual cue-induce reinstatement firstly, and then tested discrete cue-induced reinstatement. This raises the possibility that contextual cue itself might influence discrete cue-induced heroin seeking. In fact, during contextual cue phase, the rats were allowed to respond to the nose-pokes with all the conditioned stimulus lights kept off. The responses were recorded, but no consequences were produced. This phase was used to measure contextual cue- (chamber environment) induced heroin-seeking and was generally regarded as an extinction phase [50]. Furthermore, we observed that contextual cue-induced reinstatement of active responding occurred mainly in the first 15 min block and decreased across blocks. There were no significant differences in active responses between the groups in the last 15 min block.

## 5. Conclusion

EA treatment significantly reduced discrete cue-induced heroin seeking behavior in reinstatement of self-administration procedures. Fos protein expression consistent with conditioned enhancement by discrete cue stimuli was observed in the NACc core. 2 Hz EA stimulation attenuated Fos expression in the core but not the shell of the NACc. Altogether, these results support the hypothesis that EA can reduce drug-seeking and highlight the therapeutic benefit of EA in preventing relapse to drug addiction.
